# Effect of Empiric Anti–*Mycobacterium tuberculosis* Therapy on Survival Among Human Immunodeficiency Virus–Infected Adults Admitted With Sepsis to a Regional Referral Hospital in Uganda

**DOI:** 10.1093/ofid/ofz140

**Published:** 2019-03-14

**Authors:** Riley H Hazard, Peninah Kagina, Richard Kitayimbwa, Keneth Male, Melissa McShane, Dennis Mubiru, Emma Welikhe, Christopher C Moore, Amir Abdallah

**Affiliations:** 1 University of Melbourne, School of Population and Global Health, Australia; 2 Mbarara University of Science and Technology, Department of Medicine, Uganda; 3 Temple University, Department of Medicine, Division of Hematology and Oncology, Philadelphia, Pennsylvania; 4 University of Virginia, Department of Medicine, Division of Infectious Diseases, Charlottesville; 5 Mayo Clinic Phoenix, Department of Neurology, Arizona

**Keywords:** Africa, *Mycobacterium tuberculosis*, sepsis, Uganda

## Abstract

**Background:**

*Mycobacterium tuberculosis* is the leading cause of bloodstream infection among human immunodeficiency virus (HIV)–infected patients with sepsis in sub-Saharan Africa and is associated with high mortality rates.

**Methods:**

We conducted a retrospective study of HIV-infected adults with sepsis at the Mbarara Regional Referral Hospital in Uganda to measure the proportion who received antituberculosis therapy and to determine the relationship between antituberculosis therapy and 28-day survival.

**Results:**

Of the 149 patients evaluated, 74 (50%) had severe sepsis and 48 (32%) died. Of the 55 patients (37%) who received antituberculosis therapy, 19 (35%) died, compared with 29 of 94 (31%) who did not receive such therapy (odds ratio, 1.34; 95% confidence interval [CI], .56–3.18; *P* = .64). The 28-day survival rates did not differ significantly between these 2 groups (log-rank test, *P* = .21). Among the 74 patients with severe sepsis, 9 of 26 (35%) who received antituberculosis therapy died, versus 23 of 48 (48%) who did not receive such therapy (odds ratio, 0.58; 95% CI, .21–1.52; *P* = .27). In patients with severe sepsis, antituberculosis therapy was associated with an improved 28-day survival rate (log-rank test *P* = .01), and with a reduced mortality rate in a Cox proportional hazards model (hazard ratio, 0.32; 95% CI, .13–.80; *P* = .03).

**Conclusions:**

Empiric antituberculosis therapy was associated with improved survival rates among patients with severe sepsis, but not among all patients with sepsis.

## Teaser

In hospitalized HIV-infected patients in Uganda, empiric antituberculosis therapy was associated with improved survival among patients with severe sepsis.

In sub-Saharan Africa, sepsis is a leading cause of disease and death [1]. Approximately 65%–85% of all patients with sepsis are human immunodeficiency virus (HIV) infected, and *Mycobacterium tuberculosis* is the leading cause of bacteremia in this population, with infection rates of 25%–40% and an associated mortality rate as high as 50% [2–11]. Compared with persons without HIV infection, HIV-infected patients are 3–4 times more likely to die of tuberculosis sepsis [12]. Factors associated with poor outcomes in HIV-infected patients with tuberculosis sepsis include immunosuppression, malnutrition, late clinical presentation, and delayed diagnosis and treatment of tuberculosis [5, 13].

Reduction of sepsis-associated mortality rates relies on early detection and prompt initiation of antimicrobial therapy [14]. Sepsis has a similar clinical phenotype regardless of the underlying pathogen, and the lack of rapid and effective diagnostic tools for tuberculosis can lead to significant delays in the initiation of appropriate therapy and thus poor outcomes [15, 16]. Furthermore, tuberculosis in the setting of HIV coinfection is often disseminated rather than focal, which is difficult to detect microbiologically [4, 5, 17].

Culture is the reference standard for the microbiological detection of tuberculosis but is expensive, labor intensive, and has a long turnaround time, making it impractical for clinical decision making in patients with sepsis. Sputum smear microscopy and polymerase chain reaction–based platforms, such as the GeneXpert MTB/RIF platform, are faster and increasingly available but patients with sepsis rarely expectorate sputum, and sensitivity is diminished in disseminated or paucibacillary tuberculosis [18, 19]. Urinary lipoarabinomannan (LAM) testing has a fast turnaround time and can be used at the point of care to guide antituberculosis therapy but has limited sensitivity, particularly in HIV-infected patients with CD4^+^ T-cell counts >200/μL [20–22]. For patients who are sputum smear negative and without confirmed tuberculosis, World Health Organization (WHO) algorithms are available to guide clinicians in the initiation of antituberculosis therapy, but these diagnostic algorithms lack sensitivity and specificity [23, 24].

Given that tuberculosis is the leading cause of sepsis in areas with a high prevalence of HIV and tuberculosis, such as Uganda, and given the high mortality rates associated with tuberculosis sepsis and the lack of available rapid and reliable tuberculosis diagnostic tests, we aimed to (1) determine the proportion of HIV-infected patients admitted with sepsis who received antituberculosis therapy during their hospitalization, (2) identify predictors of receiving antituberculosis therapy and, (3) determine the relationship between empiric antituberculosis therapy and 28-day survival rates.

## MATERIALS AND METHODS

### Study Site

We conducted this retrospective cohort study at the Mbarara Regional Referral Hospital, located in southwestern Uganda. The national HIV prevalence is 6%, and the incidence of tuberculosis infection is 201 per 100 000 population [25, 26]. The 1200-bed hospital serves as the teaching hospital for the Mbarara University of Science and Technology. Adult patients with sepsis are initially admitted to the emergency ward, where they are resuscitated and stabilized before being transferred to the medical ward, or the tuberculosis ward if tuberculosis has been microbiologically confirmed.

Owing to resource limitations, radiographs are obtained infrequently. Laboratory test availability is also limited, so full blood counts, CD4 cell counts, and HIV viral loads are not routinely performed. During the time of the study, smear microscopy was the standard method of tuberculosis diagnosis, and neither the GeneXpert MTB/RIF nor the urinary LAM assay were in routine use. Clinicians typically follow the WHO 2007 guidelines for the management of smear-negative tuberculosis [27].

### Study Procedures

Using the medical ward registration log, we reviewed all cases of patients admitted with infection to the medical ward between January 2014 and December 2015 and analyzed data from all HIV-infected individuals who were admitted with sepsis. Our study preceded the new Sepsis-3 definitions, so we defined sepsis according to Sepsis-2 definitions: (1) clinical suspicion of infection and (2) ≥2 systemic inflammatory response criteria, defined as an axillary temperature ≥38°C or <36°C, heart rate >90/min, respiratory rate >20/min, or white blood cell count <4000/µL or >12 000/µL [28]. We defined severe sepsis as sepsis plus clinical evidence of end organ dysfunction ,including systolic blood pressure ≤90 mm Hg, mean arterial pressure <65 mm Hg, or Glasgow coma scale (GCS) score <15 [28, 29].

We included only patients with ≥1 valid recording of heart rate, respiratory rate, systolic blood pressure, diastolic blood pressure, and temperature. We used the first available recorded vital sign obtained in the emergency or medical ward to impute any missing admission vital signs. We also required included patients to have a valid discharge or death date and a valid date of antituberculosis therapy administration if it was given. We excluded patients who had microbiologically proved tuberculosis or had received antituberculosis therapy before admission. For all included patients, we obtained the following data: clinical history, vital signs, examination findings, diagnostic investigations, antituberculosis therapy, and the in-hospital outcomes of death or discharge.

We performed sensitivity analyses using different sepsis definitions including the quick sepsis-related organ failure assessment (qSOFA) and WHO tuberculosis danger signs. For qSOFA, we defined sepsis as ≥2 of the following: GCS score <15, respiratory rate ≥22/min, or systolic blood pressure ≤ 100 mm Hg [30]. For WHO danger signs, we defined sepsis as ≥1 of the following: respiratory rate >30/min, temperature >39°C, heart rate >120/min, or inability to walk unaided [27]. We did not have data regarding ability to ambulate so we substituted GCS score <15 for the criterion of inability to walk unaided. We used the universal vital assessment score to determine severity of illness [31].

### Analyses

We used the χ^2^ test to compare proportions and the Mann-Whitney *U* test to compare continuous variables. We determined clinical associations with receipt of antituberculosis therapy using multivariable logistic regression. We compared the 28-day survival probability between groups who received antituberculosis therapy and those who did not, using the log-rank test. We censored patients at the time of death, and because we did not have postdischarge mortality data, we also censored patients at the time of hospital discharge. We used Cox proportional hazards regression to determine associations with in-hospital deaths. For both regression models, we included all factors that we believed *a priori* to be clinically relevant to the outcome of interest and for which we had sufficient data. We assessed interactions using the *F* test. We also assessed the assumption of proportional hazards for the Cox model. We considered a significance level of < 0.05 to be statistically significant. We used R software R version 3.4.4 (2018-03-15) (R Foundation for Statistical Computing) for all analyses.

### Ethical Considerations

This study was approved by the institutional review boards of Mbarara University of Science and Technology and the University of Virginia.

## RESULTS

### Patient Characteristics

**Figure 1.**
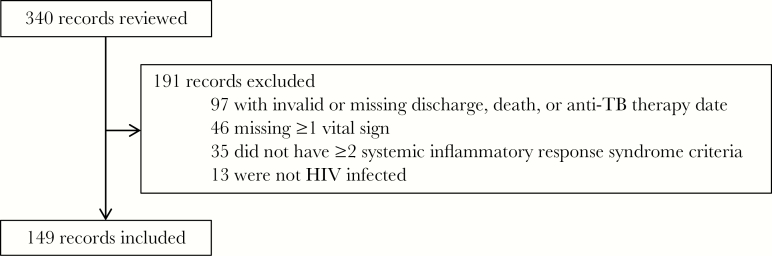
Flowchart of study inclusion among human immunodeficiency virus (HIV)–infected patients with sepsis admitted to Mbarara Regional Referral Hospital from January 2014 to December 2015.

We screened 340 patients and included 149 in our analyses (Figure 1). Among the 149 patients included, the median (interquartile range [IQR]) age was 33 (28–40) years, and 67 (45%) were women (Table 1). The median (IQR) temperature and systolic blood pressure were 38.2°C (36.8°C–38.7°C) and 98 (90–110) mm Hg. Fever, cough, shortness of breath, headache, and diarrhea were the presenting symptoms in 105 (70%), 102 (68%), 51 (34%), 39 (26%), and 33 (34%) of the patients, respectively. Recent CD4 cell counts were available for only 50 of 149 patients (34%), and the median (IQR) count was 175/μL (68–338/μL). At the time of admission, 89 of 149 patients (60%) were receiving antiretroviral therapy. Only 23 of 149 (15%) had a chest radiograph obtained during their hospitalization. Antibiotics were administered to 129 of 149 patients (87%), and 94 of 129 (73%) received intravenous ceftriaxone. Oxygen therapy was administered to 44 of 149 (30%). Of the 74 patients with severe sepsis, 19 (26%) fulfilled more than a single criterion for severe sepsis, 34 (46%) had only a GCS score <15, 18 (24%) had only a systolic blood pressure <90 mm Hg, and 3 (4%) had only a mean arterial pressure <65 mm Hg.

**Table 1.** Clinical Characteristics of Human Immunodeficiency Virus–Infected Patients With Sepsis or Severe Sepsis Admitted to Mbarara Regional Referral Hospital (January 2014 to December 2015)

Variable	All Patients (n = 149)	Patients With Severe Sepsis (n = 74)
Age, median (IQR), y	33 (28–40)	35 (29–40)
Female sex, no. (%)	67 (45)	32 (58)
Vital signs, median (IQR)		
Temperature, °C	38.2 (36.8–38.7)	38.3 (36.7–38.8)
Respiratory rate, breaths/min	30 (24–36)	28 (24–36)
Heart rate, beats/min	118 (102–126)	118 (101–124)
Systolic blood pressure, mm Hg	98 (90–110)	90 (80–100)
Diastolic blood pressure, mm Hg	60 (60–70)	60 (50–70)
Glasgow coma scale (score), median (IQR)	15 (14–15)	14 (13–15)
Cough, no. (%)	102 (68)	48 (65)
Shortness of breath, no. (%)	51 (34)	19 (26)
Treatment, no. (%)		
Antituberculosis therapy	55 (37)	26 (35)
Antibiotics	129 (87)	63 (74)
Oxygen	44 (30)	27 (37)
Blood transfusion	42 (29)	18 (25)
Death, no. (%)	48 (32)	32 (43)

Abbreviation: IQR, interquartile range.

### Antituberculosis Therapy

Antituberculosis therapy was administered to 55 of 149 (37%) patients, including 26 of the 74 (35%) with severe sepsis. The median (IQR) time to initiation of antituberculosis therapy was 4 (1–7) days for the overall cohort and 5.5 (1–8) days for patients with severe sepsis. In both univariable and multivariable analyses, we found no demographic, clinical, or treatment characteristics that were associated with the receipt of antituberculosis therapy for the total cohort or the subgroup with severe sepsis (Table 2).

**Table 2.** Adjusted Odds of Receiving Antituberculosis Therapy Among Human Immunodeficiency Virus–Infected Patients With Sepsis or Severe Sepsis Admitted to Mbarara Regional Referral Hospital (January 2014 to December 2015)

Variable	All Patients (n = 149)		Patients With Severe Sepsis (n = 74)	
	Adjusted OR (95% CI)	*P* Value	Adjusted OR (95% CI)	*P* Value
Age (y)	1.00 (.96–1.04)	.99	1.01 (.96–1.08)	.62
Female sex	0.59 (.28–1.21)	.16	0.78 (.22–2.66)	.70
Temperature (°C)	0.90 (.70–1.16)	.42	1.03 (.72–1.50)	.86
Respiratory rate (breaths/min)	0.99 (.95–1.03)	.56	0.94 (.87–1.00)	.08
Heart rate (beats/min)	1.00 (.98–1.02)	.87	0.99 (.96–1.02)	.50
Systolic blood pressure (mm Hg)	0.99 (.96–1.02)	.47	0.99 (.95–1.04)	.74
Diastolic blood pressure (mm Hg)	1.01 (.98–1.04)	.56	1.01 (.96–1.06)	.72
Glasgow coma scale score	0.94 (.70–1.29)	.71	0.91 (.63–1.32)	.61
Receipt of oxygen	0.74 (.31–1.71)	.48	0.43 (.12–1.46)	.18
Receipt of blood transfusion	1.59 (.72–3.50)	.25	1.32 (.36–4.72)	.67
Severe sepsis	0.86 (.33–2.18)	.75	…	…

Abbreviations: CI, confidence interval; OR, odds ratio.

### Sepsis

**Figure 2.**
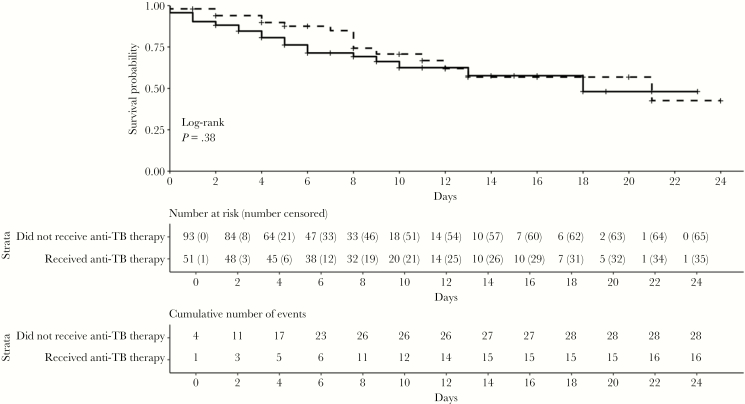
Survival curves over 28 days for human immunodeficiency virus–infected patients with sepsis admitted to Mbarara Regional Referral Hospital from January 2014 to December 2015, according to whether they received (*dashed line*) or did not receive (*solid line*) antituberculosis therapy. Patients were censored at death and at hospital discharge.

Of the 149 patients with sepsis evaluated, 48 (32%) died (Table 3). Of the 55 (37%) who received antituberculosis therapy, 19 (35%) died, compared with 29 of 94 (31%) who did not receive such therapy (odds ratio, 1.34; 95% confidence interval [CI], .56–3.18; *P* = .64). The mean interval between admission and receipt of antituberculosis therapy was 6.6 days in those who died and 4.5 days in those who survived to discharge (*P* = .13). The 28-day survival rates did not differ significantly between patients who did and those who did not receive antituberculosis therapy (log-rank test, *P* = .21) (Figure 2).

**Table 3.** Adjusted Hazard Ratios for Mortality Among Human Immunodeficiency Virus–Infected Patients With Sepsis or Severe Sepsis Admitted to Mbarara Regional Referral Hospital (January 2014 to December 2015)

Variable	Adjusted HR (95% CI)	*P* Value
Age (years)	1.01 (.97–1.04)	.74
Female sex	0.76 (.39–1.47)	.41
Temperature (°C)	0.82 (.66–1.02)	.08
Respiratory rate (breaths/min)	1.01 (.97–1.05)	.54
Heart rate (beats/min)	1.01 (.99–1.02)	.41
Systolic blood pressure (mm Hg)	0.98 (.96–1.00)	.85
Diastolic blood pressure (mm Hg)	1.01 (.98–1.03)	.63
Glasgow coma scale (score)	0.64 (.53–.78)	<.01
Receipt of oxygen	3.13 (1.63–5.99)	<.01
Receipt of blood transfusion	1.00 (.49–2.03)	>.99
Empiric antituberculosis therapy		
Patients with sepsis	1.24 (.53–2.90)	.63
Patients with severe sepsis	0.32 (.13–.80)	.03

Abbreviations: CI, confidence interval; HR, hazard ratio.

### Severe Sepsis

**Figure 3.**
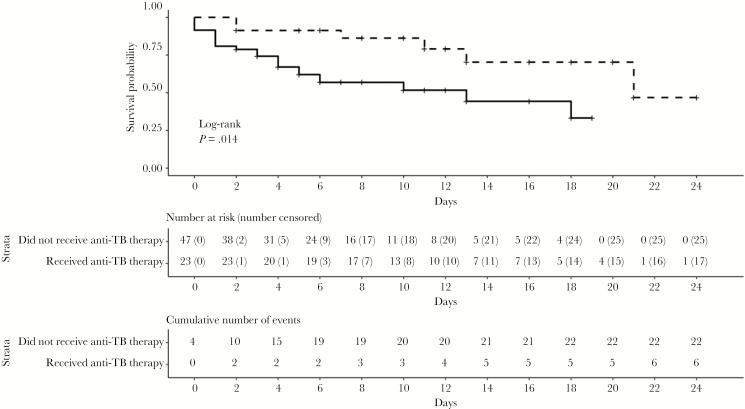
Survival curves over 28 days for human immunodeficiency virus–infected patients with severe sepsis admitted to Mbarara Regional Referral Hospital from January 2014 to December 2015, according to whether they received (*dashed line*) or did not receive (*solid line*) antituberculosis therapy. Patients were censored at death and at hospital discharge.

Among the 74 patients with severe sepsis, 9 of 26 (35%) who received antituberculosis therapy died, compared with 23 of 48 (48%) who did not receive antituberculosis therapy (odds ratio, 0.58; 95% CI, .21–1.52; *P* = .27). The mean interval between admission and receipt of antituberculosis therapy was 9.2 in patients who died and 4.6 days in those who survived to discharge (*P* = .11), respectively. Antituberculosis therapy was associated with improved 28-day survival rates (log-rank test, *P* = .01) (Figure 3). 

### Sensitivity Analyses

Among patients with sepsis, defined either by qSOFA (n = 121) or by the presence of any WHO danger signs (n = 113), There was no difference in 28-day survival rates between those who did and those who did not they receive antituberculosis therapy (log rank test, *P* = .17 and *P* = .27, respectively). However, when we modified the qSOFA blood pressure criterion for blood pressure to SBP ≤ 90 or MAP ≤ 65 mmHg, there were 86 patients who met modified qSOFA criteria and those who received empiric antituberculosis therapy had improved 28 day survival rates (log-rank test, *P* = 0.02).

### Cox Proportional Hazards Model

In the Cox proportional hazards regression model, there was a statistically significant interaction between antituberculosis therapy and severity of sepsis (*P* for heterogeneity = .03). Antituberculosis therapy was associated with a 68% reduction in mortality rate among patients with severe sepsis (hazard ratio, 0.32; 95% CI, .13–.80; *P* = .03) (Table 3). Low GCS scores (hazard ratio, 0.64; 95% CI, .53–0.78; *P* < .01) and receipt of supplemental oxygen (3.13; 95% CI, 1.63–5.99; *P* < .01) were associated with higher mortality rates (Table 3). The median (IQR) universal vital assessment score was 6 (2–8) for patients who received supplemental oxygen versus 4 (2–6) for those who did not (*P* < .01).

## Discussion

In HIV-infected patients admitted with sepsis to a regional referral hospital in southwestern Uganda, we found that antituberculosis therapy was administered to 37% of patients, including 35% of those with severe sepsis. Although empiric antituberculosis therapy was not associated with improved 28-day survival for all patients, it was associated with improved survival for those with severe sepsis. Initiation of antituberculosis therapy was delayed by up to a week after admission and we found no associations between clinical variables and the receipt of antituberculosis therapy. As in other studies of sepsis from sub-Saharan Africa, the mortality rate in our study patients was high (32%), and our findings suggest that there are opportunities to optimize clinical care of patients with sepsis, including the early administration of empiric antituberculosis therapy for patients with severe sepsis [9, 31, 32].

The WHO recommends that hospitalized patients with a history of cough for 2–3 weeks in settings with high HIV prevalence should initially be treated with parenteral antibiotics for bacterial infection, followed by antituberculosis therapy if there has been no clinical improvement after 3–5 days. In an observational before-after study in South Africa, WHO algorithm based antituberculosis therapy was associated with improved survival compared with standard practice. Within the standard practice group, antituberculosis therapy was delayed compared with the algorithm group, but it was also associated with improved survival [33]. These data suggest that there is additional survival benefit accrued from early antituberculosis therapy. In an observational study of patients with tuberculosis bloodstream infection in Uganda, the 30-day mortality rate was decreased in patients who received empiric antituberculosis therapy at admission compared with those who did not (31% vs 53%) [6]. In another study from Uganda, patients with suspected pulmonary tuberculosis and clinical danger signs who received WHO algorithm–based antituberculosis treatment had a 44% reduction in 8-week mortality [34].

Delayed antimicrobial treatment of sepsis and septic shock is associated with increased mortality rates [14]. In a study of patients with septic shock due to tuberculosis from North America and Saudi Arabia, the mortality rate was 63% for patients who received early antituberculosis therapy compared with 93% for patients who did not, and all patients who did not receive any antituberculosis therapy died [15]. However, to our knowledge no prior studies from sub-Saharan Africa have evaluated empiric antituberculosis therapy in patients with undifferentiated sepsis in whom a clinical history of cough may not be available and in whom tuberculosis may not initially be the leading clinical diagnosis.

In our study, the mean time to antituberculosis therapy was 4 days for the total cohort and 5.5 days for patients with severe sepsis. As seen in our survival curves, this is when the majority of deaths occurred, particularly for those with severe sepsis. The burden of infection, as measured by quantities of tuberculosis DNA in blood in patients with tuberculosis bacteremia, is correlated with mortality rate [9]. Therefore, delays in antituberculosis therapy may lead to an increasing burden of infection, organ failure, and in some cases an inability to rescue patients once antituberculosis therapy is initiated. Our data suggest that antituberculosis therapy should be immediately considered in all severely ill patients in similar settings, whether or not tuberculosis is initially clinically considered.

In a 2018 randomized study, the use of urinary LAM testing to guide antituberculosis therapy in hospitalized HIV-infected patients in Zambia and South Africa did not reduce the overall mortality rate in all patients but did reduce the mortality rate in a subgroup of high-risk patients [20]. This is an encouraging finding suggesting that rapid and accurate tuberculosis diagnostics could improve outcomes. However, in most clinical settings in sub-Saharan Africa, diagnostic evaluations, including urinary LAM testing, molecular tests, and blood cultures, are not available. Furthermore, urinary LAM testing is not 100% sensitive for tuberculosis bacteremia, and its sensitivity decreases as CD4 cell counts increase [20–22]. Therefore, LAM testing may miss some patients with tuberculosis, particularly those with CD4 cell counts >100/μL. Accordingly, early empiric antituberculosis therapy may be a successful strategy for the treatment of undifferentiated sepsis in settings with high prevalence of HIV and tuberculosis, particularly where diagnostic interventions are limited.

It is possible that antituberculosis therapy failed owing to pharmacokinetic limitations in some patients with sepsis or severe sepsis from tuberculosis in our study. In such critically ill patients, gastrointestinal dysfunction may lead to decreased absorption of medications, tissue hypoperfusion may lead to decreased antibiotic tissue concentrations, and hepatic dysfunction may lead to decreased protein binding, all of which can lead to underdosing in antituberculosis therapy [35]. There are no published pharmacokinetic studies of antituberculosis therapy in patients with sepsis, but in patients from South Africa with tuberculosis, low drug concentrations were associated with poor long-term outcomes of microbiologic failure, death, or relapse [36]. Drug-resistant tuberculosis could also render standard antituberculosis therapy ineffective; however, multidrug-resistant tuberculosis remains uncommon in Uganda [37]. Finally, given the limited diagnostic capacity at Mbarara Regional Referral Hospital, antituberculosis therapy may have been inappropriate for the underlying infection and may explain why empiric antituberculosis therapy was not a successful strategy for all patients in our study.

There were several limitations to this real-world study of empiric antituberculosis therapy in patients with a sepsis in a setting with high HIV and tuberculosis prevalence. First, this was a retrospective study with data missing for some patients, which could have affected our findings; however, we believe missing data occurred randomly and did not lead to bias regarding diagnosis, treatment, or outcome in the patients. Furthermore, we included only patients with complete or imputed data, so they were likely to have true sepsis. Second, GeneXpert and urinary LAM testing, recommended for use by WHO in 2010 and 2015, respectively, were not in routine use during our study [38, 39]. We also did not have stored samples for subsequent molecular or other diagnostic testing. Therefore, we were unable to microbiologically confirm the diagnosis of tuberculosis. This additional information may have allowed us to determine whether poor outcomes after antituberculosis therapy were due to a misdiagnosis or a combination of disease burden and human and material resource limitations. We mitigated possible misclassification bias by our sensitivity analyses that included alternative sepsis definitions. Finally, we collected data from patients admitted over a calendar year, and our total cohort size may have been underpowered to determine a difference in outcomes between all patients who did or did not receive empiric antituberculosis therapy for sepsis.

In conclusion, in HIV-infected patients admitted with sepsis to a regional referral hospital in Uganda, we found that 37% of patients received empiric antituberculosis therapy. Although empiric antituberculosis therapy was not associated with improved 28-day survival for all patients, it was associated with improved survival for patients with severe sepsis. Our findings are similar to other studies that suggested a benefit to early empiric antituberculosis therapy for patients with clinically suspected tuberculosis [14, 15, 34]. Because there was a considerable delay (up to a week) in administration of antituberculosis therapy for the majority of patients in our study, and given the diagnostic limitations that exist in Mbarara Regional Referral Hospital and similar hospitals throughout sub-Saharan Africa, immediate rather than delayed empiric antituberculosis therapy, as suggested by WHO may be a good strategy for treating patients with sepsis in areas with high HIV and tuberculosis prevalence. The best way to answer this question would be through a randomized controlled clinical trial.
